# FUT8 Alpha-(1,6)-Fucosyltransferase in Cancer

**DOI:** 10.3390/ijms22010455

**Published:** 2021-01-05

**Authors:** Kayla Bastian, Emma Scott, David J. Elliott, Jennifer Munkley

**Affiliations:** Institute of Biosciences, Newcastle University, Newcastle Upon Tyne NE1 3BZ, UK; Emma.Scott@newcastle.ac.uk (E.S.); david.elliott@newcastle.ac.uk (D.J.E.); jennifer.munkley@ncl.ac.uk (J.M.)

**Keywords:** fut8, cancer, glycosylation, fucosylation

## Abstract

Aberrant glycosylation is a universal feature of cancer cells that can impact all steps in tumour progression from malignant transformation to metastasis and immune evasion. One key change in tumour glycosylation is altered core fucosylation. Core fucosylation is driven by fucosyltransferase 8 (FUT8), which catalyses the addition of α1,6-fucose to the innermost GlcNAc residue of N-glycans. FUT8 is frequently upregulated in cancer, and plays a critical role in immune evasion, antibody-dependent cellular cytotoxicity (ADCC), and the regulation of TGF-β, EGF, α3β1 integrin and E-Cadherin. Here, we summarise the role of FUT8 in various cancers (including lung, liver, colorectal, ovarian, prostate, breast, melanoma, thyroid, and pancreatic), discuss the potential mechanisms involved, and outline opportunities to exploit FUT8 as a critical factor in cancer therapeutics in the future.

## 1. Glycosylation in Cancer

Glycosylation is the enzymatic process that produces glycosidic linkages of saccharides to other saccharides, proteins or lipids. It is one of the most commonly occurring post-translational modifications and one of the most complex [1,2,3]. Aberrant glycosylation is an established hallmark of cancer that plays a critical role in tumour biology [1,4,5,6,7]. Changes to glycans in cancer were initially described more than 50 years ago [8], and since then technological advances have highlighted cell-surface glycans and changes in glycosylation patterns as key drivers of cell signalling, tumour immunology, metastasis, and how tumours respond to therapy [1,4,9,10,11,12,13]. A wide range of alterations to glycans have been observed in cancer, and tumour cells consistently express specific changes to glycan epitopes, including truncated O-glycans, altered N-glycan branching, increased sialylation (the addition of sialic acid to the terminal end of glycoproteins) and increased fucosylation [4,14,15]. These modifications to glycans can be driven by the altered expression of glycosyltranserases in cancer cells.

## 2. Fucosylation

Fucosylation is a type of glycosylation, which results in the attachment of a fucose residue to N-glycans, O-glycans, and glycolipids. Fucosylation can be divided into terminal and core fucosylation. Fucosylated glycans are synthesized by a range of fucosyltransferases (Fut), of which 13 have been identified in the human genome (Table 1) [16,17]. FUT8 is unique in that it is the only Fut responsible for core-fucosylation on N-glycoproteins (as most of the other fucosyltransferases are functionally redundant this makes core fucosylation unique) [16,18,19,20].

### Core Fucosylation

**Figure 1:**
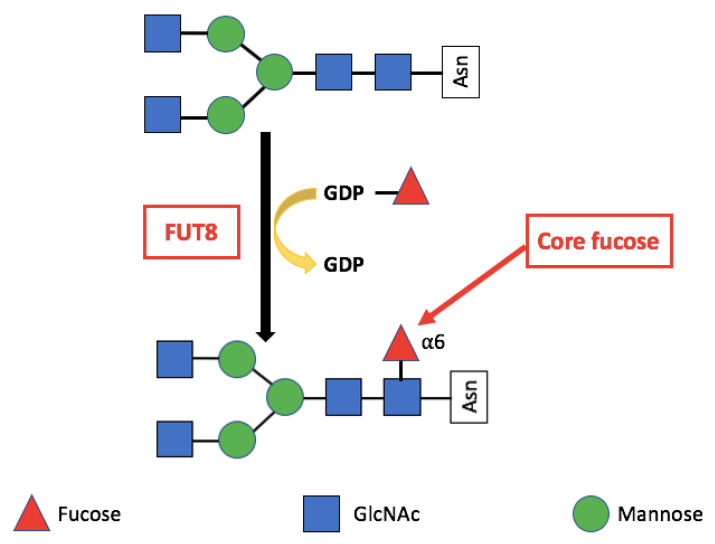
The reaction catalysed by FUT8. FUT8 transfers an l-fucose reside from GDP-β-l-fucose (GDP-Fuc) onto the innermost GlcNAc of an N-glycan to form an α-1,6 linkage.

**Figure 2:**
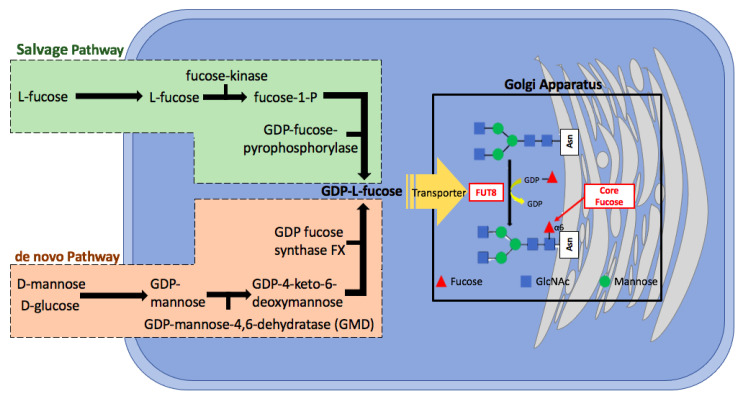
The sythetic pathway for GDP-fucose. GDP-fucose, an essential component of core fucosyaltion is produced by two different pathways within the cell. The predominat de novo pathway relies on GMD and FX proteins. Adapted from [32].

FUT8 mediated core fucosylation is an important post-translational modification, that has been linked to cancer cell invasion and metastasis [24,25,26,27,28,29,30,31]. In the Golgi apparatus FUT8 transfers an l-fucose reside from guanosine diphosphate (GDP-β-l-fucose) (GDP-Fuc) onto the innermost GlcNAc of an N-glycan to form an α-1,6 linkage (Figure 1). Core fucose levels can be affected by the level of GDP-Fuc substrate and/or its transport into the Golgi [16,32,33]. GDP-fuose is generated in the cytosol by two distinct pathways from l-fucose, the more dominate de novo synthesis and the salvage pathway (Figure 2). The de novo pathway transforms GDP-mannose to GDP-fucose via three enzymatic reactions carried out by two proteins, GDP-mannose 4,6-dehydratase (GMD) and a second enzyme, GDP-keto-6-deoxymannose 3,5-epimerase, 4-reductase (otherwise referred to as the FX protein) [3,34]. The de novo pathway accounts for 90% of GDP-fucose synthesis and was discovered over 40 years ago. In contrast the salvage pathway synthesizes GDP-fucose from free fucose derived from extracellular or lysosomal sources [16].

## 3. Alpha-(1,6)-Fucosyltransferase (FUT8)

The Fucosyltransferase 8 (FUT8) gene is located on chromosome 14q23.3 and encodes an enzyme that catalyses core fucosylation—an essential N-glycan modification [35]. FUT8 is the only enzyme responsible for core-fucosylation on N-glycoproteins [19]. It is expressed in various human tissues with particularly high levels in the brain, placenta, lung, stomach, and small intestine [36]. 

It is worth noting the physiological role of FUT8 is crucial: homozygous knockout mice experience early postnatal death, severe growth retardation and emphysema-like changes in the lung, suggesting the importance of this fucose modification for growth factor receptor activation [37]. No oligosaccharide structures with core fucose are present in FUT8 knockout mice, and 70–80% of mice die two to three days after birth. This is in contrast to the other FUT enzymes, for which loss of function does not cause lethality [18,32,38,39].

### Structure

Several glycosyltransferases have been crystallographically analysed to identify two structural superfamilies (GT-A and GT-B) [40]. GT-A enzymes have the DXD or EXD motif, containing a Rossman fold (a nucleotide-sugar binding domain) of two tightly associated domains at the N-terminus. GT-B enzymes have folds involving two similar Rossman folds [41,42]. Even though FUT8 contains one Rossman fold, the catalytic site of FUT8 lacks the characteristic elements typical of GT-A enzymes. FUT8 is thought to have a catalytic region closer to GT-B enzymes. Similar to those of GT-B enzyme, FUT8 also has a DXD motif and is fully active without a metal ion.

**Figure 3:**
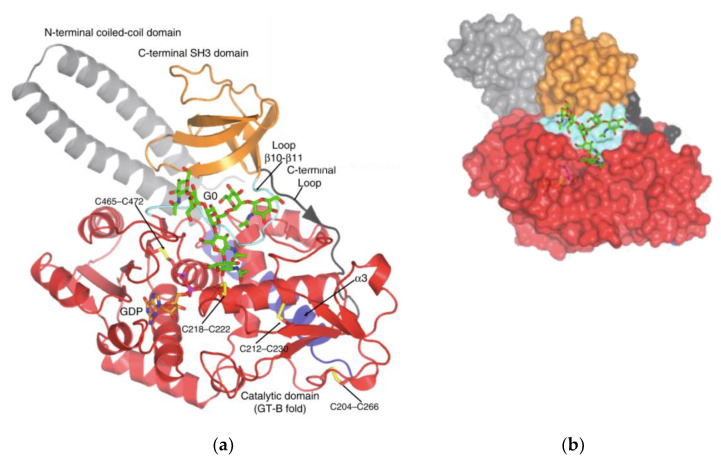
Structure of FUT8. Published by [43] and reproduced with permission. (**a**) Ribbon structure of HsFUT8 with orange carbon atoms representing GDP and green carbon atoms representing a bi-antennary complex N-glycan (G0). The coiled-coil domain is colored in gray, the catalytic domain in red, and the SH3 domain in orange. The interdomain α3 and loop β10–β11 are colored in blue and aquamarine, respectively. Yellow sulfur atoms indicate disulfide bridges. The C-terminal loop is colored in black. Electron density maps are F_O_–F_C_ (blue) contoured at 2.2σ for GDP and G0. (**b**) Surface representation of the HsFUT8-GDP-G0 complex.

FUT8 is a multi-domain enzyme containing an N-terminal coiled-coil domain, a catalytic domain (which adopts a GT-B fold), and a luminal C-terminal Src homology 3 (SH3) domain revealed by the crystal structure (Figure 3) [40]. The acceptor specificity of FUT8 requires that a terminal GlcNAc moiety on the α1,3 arm of the N-glycan is present, with more flexibility on the α1,6 arm [43]. A peptide/protein is not required unless FUT8 is fucosylating a high mannose N-glycan lacking a GlcNAc moiety, in which case a peptide/protein is required [43]. For many years the reaction mechanism for FUT8 remained elusive. Recently, it was discovered that the FUT8 structure allows for conformational changes similar to a SN2 single displacement reaction mechanism. What differentiates FUT8 is that instead of an amino acid acting as a catalytic base already present in the binding site in the absence of ligands, the catalytic base is brought to the binding site in the presence of ligands. It is with this newly provided information on the structure and enzymatic mechanism of FUT8 that the development of inhibitors could be developed for cancer therapy.

The SH3 domain is typical of signal transduction molecules in the cytosol, but is unique to FUT8 compared to other glycosyltransferases (and likely plays are an important role in FUT8’s activity and core fucosylation) [44]. FUT8 is mainly localized to the Golgi with a type II topology, however, Tomida et al. have reported that FUT8 can be partially localized to the cell surface in an SH3-dependent manner [44,45]. Amino acid His535 in the SH3 domain is an essential residue for the enzymatic activity of FUT8, and ribophorin I (RPN1) has been identified as an SH3-dependent binding protein of FUT8, that can stimulate core fucosylation [44]. 

## 4. FUT8 Expression in Cancer

Aberrant fucosylation is one of the most important oligiosaccharide modifications involved in cancer and inflammation and is often caused by dysregulated expression of fucosyltransferases (FUTs) [1,12,32,46,47,48]. α1,6-fucosyltransferase, encoded by FUT8, has demonstrated involvement in biological and tumour characteristics and is upregulated in various cancers including lung [20,49], liver [50,51,52], colorectal [24], ovarian [53], prostate [54], breast [30], melanoma [55], thyroid [56], and pancreatic [27] (Table 2). FUT8 has been associated with patient outcomes, and is a suggested prognostic biomarker for patients with lung cancer [31], colorectal cancer [51] and prostate cancer [5].

Fucosylated glycoproteins hold promise as cancer biomarkers [5,32,58]. At present, there is an absence of clinically relevant biomarkers for predicting immunotherapy treatment outcomes. α_1_-Acid glycoprotein (AGP) is a major serum glycoprotein that possesses a range of immunomodulating effects and has five N-linked complex type glycan structures [59]. During periods of acute and chronic inflammation, particularly in the presence of tumours, the glycan structures on AGP change dramatically. AGP glycoforms containing highly fucosylated triantennary and tetraantennary sugar chains have been detected in the serum of patients with various advanced malignancies and are associated with poor prognosis [60]. Above normal levels of tri- and tetraantennary glycan chains in AGP (FUCAGP) have also been linked to poor prognosis in patients with esophagus, stomach, lung, breast, liver, pancreas, colon and rectum cacinomas [59,61,62]. Moreover, α1,3fucosylated AGP (fAGP) has been proposed as a marker of disease progression and prognosis in various cancers. In patients with advanced lung cancer, fAGP levels have been shown to predict a good and/or poor response to the immune checkpoint inhibitor Nivolumab [59]. Collectively, these examples emphasize the importance of fucosylated glycoproteins as potential biomarkers of cancer. 

### 4.1. FUT8 Expression in Lung Cancer

Lung cancer is the most commonly diagnosed cancer and is the leading cause of cancer death worldwide [63]. Lung cancer can be separated into two main forms which are classified by the type of cells from which the cancer originates [64]. The most common form (affecting 87% of lung cancer patients) is non-small-cell lung cancer which can be further divided into three types—squamous cell carcinoma, adenocarcinoma, and large-cell carcinoma. The less common form but more metastatic is small-cell lung cancer. FUT8 is up-regulated in non-small cell lung cancer (NSCLC) and is correlated with tumour metastasis, disease recurrence and poor survival in patients [49]. One study utilized aggressive lung cancer cell lines (CL1-5 and PC14) and found FUT8 knockdown significantly inhibited their malignant behaviours, including in vitro invasion and cell proliferation, and in vivo metastasis and tumour growth [49]. A study by Honma et al., examined the expression of FUT8 in 129 NSCLCs using immunohistochemistry and identified that over half of the samples exhibited high expression of FUT8, which was also associated with poor survival [31]. The prognostic potential of FUT8 is further supported by its significant association with unfavourable clinical outcomes in patients with potentially curatively resected NSCLCs [31]. 

Core fucosylation is needed for the proper function of the TGF-β receptor and thus TGF-β–induced epithelial-mesenchymal transition (EMT) [49,65]. In support of this, FUT8 has been shown to be up-regulated during EMT in several cancers suggesting a positive feedback loop that promotes EMT and tumour development [49]. A proposed molecular mechanism linking FUT8 and lung cancer progression involves the nuclear accumulation of β-catenin, which occurs during EMT when cancer cells lose the expression of E-cadherin. The nuclear β-catenin along with lymphoid enhancer binding factor 1 (LEF-1) activates FUT8 expression, causing FUT8 upregulation, globally altering core fucosylation, cancer cells’ response to extracellular matrix, growth factors, and other elements of the tumour microenvironment.

### 4.2. FUT8 Expression in Liver Cancer

Liver cancer is the second leading cause of cancer death [29]. The most common type of liver cancer is hepatocellular carcinoma (HCC) [52,66]. Unfortunately, the median survival of most patients after diagnosis is 6–9 months, emphasising the necessity for improved diagnostic biomarkers and targets for therapeutic treatments [52,67]. Serum α-fetoprotein (AFP) is a gold standard biomarker for the diagnosis of hepatocellular carcinoma (HCC), however the specificity of AFP for HCC is relatively low and often does not distinguish HCC from other liver diseases. Increased fucosylation is a promising marker for monitoring the progression from chronic liver disease to HCC [52], and levels of FUT8 have been shown to be increased on the cell surface and in the serum samples of HCC patients [68]. A study conducted by Egashira et al., developed and characterized a glycan antibody specific for α1-6 fucosylated AFP which could improve the specificity of AFP to improve HCC diagnosis [69]. Furthermore, when matrix-assisted laser desorption ionization-time of flight mass spectrometry (MALDI-TOF-MS) was applied to profile N-glycans in HCC tissues, FUT8 was significantly up-regulated in HCC compared to adjacent tissues [52]. Work conducted on cell lines found that expression of FUT8 is upregulated in cells with high metastatic potential [29,52]. MicroRNAs may prove valuable in inhibiting fucosylation machinery in HCC. It is believed that miR-26a, miR-34a, and miR-455-3p directly bind and negatively regulate FUT8 mRNA stability thus reducing FUT8-mediated progression of HCC and proposing a new therapeutic intervention for HCC patients [52,70]. 

### 4.3. FUT8 Expression in Colorectal Cancer

Although the number of people diagnosed with colon cancer has decreased since the mid-1980’s thanks to increased screening, colon cancer still remains the third leading cause of cancer-related deaths worldwide [71]. FUT8 was recently suggested as a direct transcriptional target of wild-type p53 in HCC cells [72], and this prompted the prediction that p53 status may affect the expression and function of FUT8 in colorectal cancer (CRC). One exploratory study, focused on the prognostic value of FUT8 expression on disease free survival (DFS) in patients with stage II and III CRC after curative surgery, found FUT8 is significantly associated with better DFS in tumours with negative p53 [51]. Contradictory findings come from studies conducted by Muinelo-Romay et al. and Noda et al. who found no association between DFS and the expression of FUT8 in their respective immunohistochemistry (*n* = 141 and *n* = 123) and microarray (*n* = 357) cohorts [51,73]. In tumours without p53 alterations, high levels of FUT8 protein expression were significantly associated with better DFS. This finding could imply that any prognostic value of FUT8 expression may be confined to the patient subgroups with p53-negative tumours. Although these studies have opposing findings, limitations such as variation in p53 detection techniques, and sample size may have influenced their results [51]. 

### 4.4. FUT8 Expression in Ovarian Cancer

Epithelial ovarian cancer (EOC) is the most lethal female genital tract cancer in the world (<30% 5-year survival rate), largely due to the 90% of patients who experience chemo-resistance [53]. The exact mechanisms responsible for chemoresistance are not fully understood, however reduced cellular accumulation of platinum-based drugs, enhanced detoxification capability, and aberrant apoptosis pathways are a few of the suggested mechanisms [53]. Studies have indicated a key role for core fucosylation in the progression of EOC [74,75].

The role of core fucosylation in the drug resistance of EOC is a focal point of research. This is due to the association of core fucosylation with TGFβ1, epidermal growth factor, and B cell receptors, as well as the uptake of drugs by cancer cells being dependent on the molecular interaction between cell surface proteins and drugs. In EOC the inhibition of cisplatin (cDDP) uptake is the main cause for cDDp-resistance. Studies show that serum and tissue from cDDP-treated EOC patients have increased levels of core fucosylation, and that the binding of Copper transporter 1 (CTR1) (an important transporter in regulating the uptake of cDDP) is supressed when core fucosylation levels are elevated [53].

### 4.5. FUT8 Expression in Prostate Cancer

Worldwide it is estimated that there is a total annual number of over 1.2 million cases of prostate cancer [76]. It is the most common male cancer in 91 countries worldwide and claims over 350,000 lives a year [77]. The highly heterogenous nature of prostate cancer leaves large gaps in diagnosis and treatment. Prostate cancer is severely lacking a method of diagnosis that is both sensitive and specific. The current biomarker PSA does not distinguish between indolent and aggressive prostate cancers and therefore does not serve as a prognostic biomarker. Decreased fucosylation on prostate-specific antigen (PSA) [78] and integrins [79], as well as elevated fucosylated haptoglobin are under investigation as potential non-invasive biomarkers for prostate cancer [80]. In a study conducted by Peracaula et al., the biochemical properties of PSA in the LNCaP cell line and from normal seminal fluid was investigated to aid in distinguishing between PSA from normal and tumour origins [58]. The difference reported in the context of fucose suggest the importance of analysing PSA glycosylation to improve its ability to distinguish between benign and malignant prostate cancer.

FUT8 drives increased core fucosylation in prostate cancer and has been linked to disease progression [5]. Overexpression of FUT8 has been positively correlated with the epithelial compartment and high grade prostate cancer [54]. In particular, higher FUT8 expression is present in high grade prostate cancer compared to low grade prostate cancer [54]. In tissue it was observed that a higher percentage of epithelial compartment (cores) were stained strongly in cases with a high grade cancer, suggesting FUT8 epithelial compartments may be associated with more aggressive disease [54]. Additionally, upregulation of FUT8 is correlated with increased fucosylation of glycoproteins in aggressive prostate cancer cells [81]. FUT8 is expressed in PC3 and DU145—prostate cancer cell lines [80], normal prostate epithelial cells (PrECs) [78], and normal prostate stromal cells (PrSCs) [78]. PC3 cells are an androgen-independent cell line with high metastatic potential, while LNCaP cells are an androgen-dependent cancer cell line [81]. It was in these two model prostate cancer cell lines that FUT8 was found to aid in the regulation of cancer cell migration [54]. Furthermore, FUT8 has been shown to be elevated at the protein level in metastatic cancer tissue in comparison to primary cancer tissue (*n* = 20) [54].

In 2018, the first study was published reporting the functional role of the FUT8 enzyme in castrate-resistant prostate cancer development. It was shown that androgen ablation is essential for FUT8 overexpression in AR-positive prostate cancer cells. Ectopic overexpression of FUT8 in androgen-dependent cells resulted in suppression of PSA production, while an increase in PSA was observed when endogenous FUT8 was knocked down. In accordance with this, patient samples with overexpressed FUT8 have been found to have lower levels of PSA [57]. In a recent study, the systemic impact of altered FUT8 on prostate cancer-derived extracellular vesicles (EVs) was determined using EV characterization and quantitative proteomics [82]. The findings revealed that the number of vesicles secreted by prostate cancer cells was reduced when the cellular expression of FUT8 was increased. In contrast the abundance of proteins associated with cell motility and prostate cancer metastasis increased when FUT8 expression is elevated [82].

### 4.6. FUT8 Expression in Breast Cancer 

Breast cancer is the most common cancer in women worldwide [83]. Even though breast cancer is heterogenous, most subtypes are hormone-related [83]. FUT8 has been linked to the migratory and invasive capabilities of aggressive breast cancer cell lines, and identified as highly upregulated in TGFβ-induced EMT [30]. FUT8 expression has been observed in normal human epithelial cells (MCF-10A), low-metastatic breast cancer cell lines (T-47D), and mesenchymal-like highly invasive breast cancer cell lines (MDA-MB-231 and Hss578T), with protein levels highest in the highly invasive breast cancer cells [30]. Furthermore, a combination of data mining of FUT8 expression at the mRNA level and the analysis of three public microarray datasets revealed high FUT8 levels are associated with cancer cell invasiveness and poor prognosis [30,84,85,86,87]. Tu et al. revealed a role for FUT8 in stimulating breast cancer cell invasion and metastasis. The research also involved a pharmacological proof-of-concept experiment using the fucosylation inhibitor, 2-fluorinated-peracetyl-fucose both in vitro and in vivo. In agreement with genetic inactivation of FUT8, administration of the fucosylation inhibitor repressed the mobility, invasiveness, and lung metastasis of breast cancer cells [30].

### 4.7. FUT8 Expression in Thyroid Cancer

There are two types of thyroid cancers papillary and follicular, with papillary being the most common [56]. Although thyroid cancer has a slow growth rate, once anaplastic the malignancy is the most rapid and progressive of all human carcinomas [56,88]. The polar characteristic of this cancer has initiated research aimed at elucidating the mechanisms responsible for the transition [56]. One of the few studies examining the role of FUT8 in thyroid cancer utilized immunohistochemistry of 133 thyroid tumours, and found FUT8 is associated with larger tumour volumes and lymph node metastasis [56]. Further studies are needed to elucidate the link between FUT8 and aggressive papillary carcinomas, identify the mechanisms involved, and understand whether FUT8 plays a role in the development of papillary carcinoma prior to anaplastic transformation [56,89]. 

### 4.8. FUT8 Expression in Melanoma

Melanoma is considered an incurable disease with incidence increasing twofold every 20 years. To date, very few systematic studies focusing on aberrant glycosylation in melanoma have been conducted. In 2017, a systems-based study by Agrawal et al. highlighted the importance of FUT8 in melanoma metastasis, and validated the criticality of core fucosylation in the adaptation of cancer cells to metastatic sites. Analysis of formalin-fixed embedded patient matched primary and metastatic melanoma tissues (*n* = 34 total) using lectin microarrays, identified higher levels of core fucose structures (α -1,6 fucose; PSA, LcH) in metastatic tumours [55]. This finding is consistent with publicly available transcriptomic data from The Cancer Genome Atlas (TCGA) [55,90,91]. Agrawal et al. also demonstrated that silencing of FUT8 in vivo reduces metastatic dissemination of melanoma cells to the lungs and inhibits the growth of pre-seeded metastases in liver, brain, and kidney. This study suggests that core fucosylation may be required for the adaptations that disseminated cancer cells undergo to survive in “foreign” tissues, highlighting FUT8 as a potential therapeutic potential for treating metastatic melanoma [55].

### 4.9. FUT8 Expression in Pancreatic Cancer

The most commonly diagnosed type of pancreatic cancer is pancreatic ductal adenocarcinoma (PDAC). Although chemotherapeutic improvements have been made, surgical resection is the only chance of cure for the disease. The overall survival time for PDAC that cannot be surgically removed is only 8.5 months. FUT8 expression is significantly higher during the development of adenoma into carcinoma compared to expression levels in normal pancreatic duct tissue [92]. High FUT8 expression is also associated with lymph-node metastasis, higher recurrence rate, and poorer prognosis. The function of FUT8 in pancreatic cancer is not fully understood. However, there are indications that FUT8 may stimulate the invasiveness and metastasis of PDAC by inducing EMT, with heavy involvement of core fucosylated TGF-β [27]. 

The results from multiple pancreatic studies highlight the pronounced elevation of fucosylated proteins in the serum of pancreatic cancer patients in comparison to patients with other cancers such as hepatocellular carcinoma, gastric cancer, and colorectal cancer [93,94]. Furthermore, in pancreatic cancer patients, site-specific fucosylation of bi-antennary glycans in two sites (N207 and N241) increased, while tri-antennary glycans (glycans containing three branches) increased in four haptoglobin N-glycan sites [93]. These findings suggest that fucosylated haptoglobin could serve as a novel marker for pancreatic cancer [93].

## 5. Molecular Mechanisms of FUT8 in Cancer

The regulatory mechanisms of FUT8 in cancer are not fully understood but important associations have been made. Studies indicate that core fucosylation can regulate the expression of programmed cell death protein 1 (PD-1) [95], and can alter antibody-dependent cellular cytotoxicity (ADCC) [43]. In addition, core fucosylation also regulates transforming growth factor- β1 receptor (TGF-β) [30], epidermal growth factor (EGF) receptor [26,96], α3β1 integrin [25], and E-cadherin [28,97,98].

### 5.1. Immune Evasion

FUT8 plays a key role in immune evasion in cancer, and has shown promise as a potential therapeutic target to improve responses to immunotherapy. Genetic ablation or pharmacological inhibition of FUT8 reduces cell-surface expression of the immune checkpoint protein PD-1, thus promoting enhanced T cell activation, and resulting in more effective tumour eradication. PD-1 contains two primary N-glycosylation sites that are heavily core fucosylated—these post-translational modifications are key regulators of PD-1 expression, and thus play a critical role in anti-tumoural response optimisation. Additionally, exhausted T cells in tumours also contain highly core-fucosylated structures including PD-1. Together these findings suggest that FUT8 can damper tumour-infiltrating immune cells, and that targeting core fucosylation may inhibit tumour growth, thus reducing PD-1/PD-L1 interactions and weakening tumour immune evasion [95].

### 5.2. Antibody-Dependent Cellular Cytotoxicity (ADCC)

ADCC is a lytic attack on antibody-targeted cells that is elicited upon binding of lymphocyte receptors (FcRs) to the constant region (Fc) of antibodies [99]. There are five types of immunoglobulin isotypes, of which immunoglobulin G (IgG) is the most abundant in human blood, and is responsible for 10–20% of plasma protein [100]. Therapeutic antibodies of human IgG1 comprised of two biantennary complex-type N-linked oligosaccharides in the constant region can mediate effector functions through the Fc and also influence ADCC [101,102]. 

Most licensed therapeutic antibodies possess core fucosylated Fc oligiosaccharides—this stems from their production in rodent mammalian cell lines. To achieve optimal ADCC therapeutic antibodies that fully lack core fucosylation can be produced [102,103]. Studies supporting the optimization of ADCC with defucosylated antibodies have primarily been conducted in T-cell leukaemia and lymphoma [104,105,106,107]. Double gene knockout of key oligiosaccharide fucose modifying genes FUT8 and GDP-mannose 4,6-dehydratase (GMD) in antibody-producing Chinese hamster ovary (CHO) cells have previously been achieved [102]. It is believed that converting already established antibody-producing cells to non-fucosylated antibody producers promotes a more potent efficacy [102,103]. Without optimizing the potential of ADCC, antibodies are administered to cancer patients at high dosages and unfortunately high costs [102]. Strategies aimed at creating defucosylated antibodies that enhance ADCC offer the potential for next-generation therapeutic antibodies. Specifically, inhibiting FUT8 activity holds promise to help promote a potent anti-tumoral response [43,102]. 

### 5.3. Transforming Growth Factor Beta (TGF-β)

Core fucosylation alters cell surface molecules and the tumour microenvironment inclusive of the extracellular matrix and growth factors, promoting the progression of cancer. The presence of core fucose greatly affects the binding affinity of TGF-β receptor toward TGF-β, making it critical to its function [20]. TGF-β has been shown to have an immunosuppressive effect in cancer, preventing anti-tumour immune mediated killing [108]. FUT8 knockdown results in lessened TGFβ-inducing EMT, while an upregulation of FUT8 is observed during EMT—suggesting a positive feedback loop between FUT8 expression and TGFβ receptor signalling to promote EMT and tumour development [30,65]. EMT is a necessary process that transforms benign tumours to aggressive and highly invasive cancer [109]. In breast cancer, upregulated FUT8 remodels TGF-β RI and RII complexes to promote downstream signalling, and genetic or pharmacological interruption of FUT8 inhibits TGF-β signalling and suppresses breast cancer metastasis in vivo [30]. 

In lung cancer cells regulation of the β-catenin/ lymphoid enhancer-binding factor-1 (LEF-1) binding (a major signaling event in EMT), is linked to FUT8 upregulation and disease progression, however in breast cancer cell studies suggest that TGF-β mediated upregulation of FUT8 is independent of β-catenin/LEF-1 [30,49]. FUT8 may be regulated by EMT-inducing transcription factors such as TWIST, SNAIL, SLUG, ZEB1, or ZEB2, with specific attention to SNAIL [30,110]. The 5’-flanking promoter region of the *FUT8* gene is comprised of several enhancer box (E-box) motifs, which may help explain the involvement of the above E-box binding proteins [111]. Although the precise structural and functional basis underlying the upregulation of FUT8 during TGF-β-induced EMT still remains under investigation, the discovery of the regulatory mechanisms could unveil promising prognostic or therapeutic leads for cancers [30]. 

### 5.4. Epidermal Growth Factor (EGF)

Core fucosylation also plays a key role in the regulation of EGF within multiple cell types. In Fut8^−/−^ embryonic fibroblast cells (derived from Fut8 null mice), EGF-induced phosphorylation of EGFR is blocked (while no significant changes in tyrosine phosphatase total activities are observed) [26]. FUT8 overexpression can also increase EGFR fucosylation to enhance EGF stimulus response and decrease sensitivity to tyrosine kinase inhibitors. When A549 cells (a human non-small cell lung cancer cell line) are depleted of FUT8, EGFR fucosylation is reduced, as is EGF-mediated cellular growth response and sensitivity. Consistent with this, overexpression of FUT8 in HEK293 cells (a human embryonic kidney cell line) increases EGF-mediated cellular growth [96].

### 5.5. α3β1. Integrin

In addition to core fucosylation deficiency being reported to down-regulate the functions of TGF-β receptor and EGF receptor, loss of core fucosylation can also inhibit α3β1 integrin-mediated cell migration and cell signalling. In Fut8^−/−^ cells integrin-mediated migration and cell signalling are decreased and can be partially rescued with the reintroduction of FUT8. Like other glycosyltransferases that play a role in the regulation of integrin functions, it is strongly suggested that FUT8 is essential for the functions of α3β1 [25].

### 5.6. E-Cadherin

Increased core fucosylation on E-cadherin is known to strengthen cell-cell adhesion and cell migratory processes via regulation of E-cadherin turnover and expression levels [24]. FUT8 and E-cadherin protein levels are significantly increased in primary colorectal cancer samples. A study conducted by Osumi et al., examined E-cadherin in FUT8 transfected human colon carcinoma cells, FUT8 knock down cells, and Fut8 deficient cells from Fut8^−/−^ mice. The results demonstrated that the activity of FUT8 regulates the total amount of E-cadherin [24]. In aggressive lung cancer, E-cadherin is oftern dysregulated. Geng et al., found that in highly metastatic lung cancer cells E-cadherin is core fucosylated while absent in lowly metastatic lung cancer cells. Furthermore, their results confirmed that E-cadherin is the substrate of FUT8 and that core fucosylated E-cadherin is positively correlated with cancer metastasis. Upregulation of FUT8 increases the levels of core fucosylation on E-cadherin and inhibits the function of E-cadherin [28,97,98].

## 6. Therapeutic Approaches

### 6.1. Afucosylated Antibodies Production Strategies

Antibody-dependent cellular cytotoxicity (ADCC) plays a critical role in tumour cell eradication. ADCC is controlled almost exclusively by the absence of fucose on IgG1, and IgG1 defucosylation can improve antibody effector function. Afucosylated anti-cancer antibodies with enhanced ADCC are expected to improve the efficacy and reduce the dose and cost of therapeutic antibodies [112,113]. Antibodies completely lacking core fucosylation can be produced in CHO cell lines containing zinc-finger nucleases (ZFNs) that cleave the *FUT8* gene [114]. In addition to CHO cells, plant cells have been considered as expression platforms for the production of recombinant antibodies [113,115].

Rat hybridoma YB2/0 cells have lower levels of *FUT8* mRNA than CHO cells and have shown promise for the production of afucosylated antibodies. Shinkawa et al. found antibodies produced in YB2/0 cells have lower levels of core fucose and can have a 50-fold higher ADCC than antibodies produced in CHO cells [99,113]. Small molecules that inhibit fucosylation (such as 2-fluorofucose and 5-alkynylfucose) can also be utilised to help produce afucosylated antibodies. These fucosylation inhibitors produce monoclonal antibodies through the proposed mechanistic actions of intracellular GDP-fucose depletion, consequently blocking the *de novo* pathway (discussed in Section 2) or through the inhibition of FUT8 [113,116]. 

### 6.2. Therapeutic Afucosylated Antibody Drugs

Various strategies have been used to produce afucosylated antibodies with improved therapeutic efficacy. To date, three afucosylated antibodies are on the market, and more than 20 are under evaluation in clinical trials [113]. Promising afucosylated antibody drugs include afucosylated Rituximab (an anti-CD20 monoclonal antibody used to treat B-cell malignancies including non-Hodgkin lymphoma), which has greater ADCC and B-cell depletion, and lower complement-dependent cytotoxicity than rituximab [117,118]. The efficacy of CD20 antibody therapy has also been improved by the development of an Fc-engineered type II humanised antibody (known as Obinutuzumab or GA101). Compared to rituximab, GA101 has a <30% reduction in fucosylation and has increased direct and immune effector cell-mediated cytotoxicity and superior antitumour activity [113,119]. GA101 has FDA approval for patients with chronic lymphocytic leukemia (CLL) or follicular lymphoma [113,119], where it promotes increased ADCC, rapid B cell removal in peripheral blood, and prolongs overall and progression-free survival in CLL patients [120,121,122].

Afucosylation has also been used to improve the efficacy of CD19 antibody drugs (which are used for therapy of B cell malignancies). CD19 is a B cell marker, and elevated levels of CD19 are detected in B cell lymphomas. Afucosylated anti-CD19 antibodies have potent B cell depletive activity and inhibit lymphoma growth in vivo [123,124,125,126,127]. Antibodies with reduced fucosylation have also been produced against EGFR, insulin-like growth factor 1 and c-Met. These antibodies have shown enhanced in vivo efficacy and tolerability in animal models and have progressed to human clinical trials [113,128,129,130]. 

Other notable afucosylated antibodies include mogamulizumab (POTELIGEO^®^), Ublituximab (TG-1101), TrasGEX (GT-MAB7.3-GEX, Glycooptimized Trastuzumab-GEX) and SEA-CD40. Mogamulizumab is an afucosylated monoclonal antibody that selectively binds to CC chemokine receptor 4 (CCR4). Mogamulizumab is approved in Japan for the treatment of hematologic malignancies and cutaneous T-cell lymphoma (CTCL) [113]), and is in multiple clinical trials (in combination with other drugs) for the treatment of solid tumours, and also for the treatment of human T-lymphotrophic virus 1 (HTLV1)-associated myelopathy [113,131]. Ublituximab (an afucosylated monoclonal antibody that targets a unique epitope on CD20 exprrssing B cells) has been tested in clincal trials for patients with B-cell non-Hodgkin lymphoma or CLL who were previously treated with rituximab [132,133]. TrasGEX (a glyco-optimised anti-HER2 antibody with enhanced ADCC) can induce longstanding remission in HER2+ relapsed metastatic colon cancer patients [134], and SEA-CD40 (a non-fucosylated anti-CD40 antibody) has potent pharmacodynamic activity in preclinical models and patients with advanced solid tumors [135,136,137].

### 6.3. Fucosylation Inhibitor-2-Fluorofucose

A number of fucosyltransferases inhibitors have been developed employing a variety of strategies. One common approach has been mimicking the natural substrate guanosine diphosphate fucose (GDP-Fuc), while others have developed fucose derivatives metabolized through the salvage pathway, that target fucosyltransferases by competitive inhibition [138]. 2-fluorofucose (SGN-2FF) is one inhibitor that acts via the salvage pathway and has shown promising anticancer effects both in vitro and in vivo [138]. SGN-2FF is a small molecule inhibitor of fucosylation with direct and indirect effects on immune cells, tumour cells, and the tumour microenvironment [139]. The inhibitor is passively transported over the cell membrane, deprotected by esterases, and metabolized to its corresponding GDP-analog [140]. Fucosylation is decreased through feedback inhibition of de novo biosynthesis, as well as competitive inhibition of fucosyltransferases [140]. SGN-2FF is an orally bioavailable inhibitor that has been used in various mouse models [30,116,141,142]. In one study, tumours were implanted in multiple strains of SGN-2FF treated mice to determine how differences in the immune repertoire affect the antitumour activity. It was found that in mice with intact immune systems SGN-2FF is reliant on T cell activity. T cells isolated from SGN-2FF-treated tumour bearing mice were transferred to naïve tumour-bearing mice, delaying tumour growth. The same effect was not observed when T cells from untreated tumour-bearing mice were isolated. The results suggest that the activity of SGN-2FF is influenced by afucosylated immune cells [139].

SGN-2FF has been tested in a phase 1 multicentre clinical trial for patients with advanced solid tumours (NCT# 02952989) [113,143]. Preliminary data supported the biological effects of SGN-2FF. Data suggested that fucosylation on the cell surface of granulocytes, and IgG fucosylation was significantly decreased, while neutrophil count was significantly increased [139]. In addition to the antitumor activity of SGN-2FF as a monotherapy, the study aimed to assess how the drug acts when in combination with the standard approved dose of Pembrolizumab (anti-PD-1) [113,143]. The study had to be terminated after three years due to overall benefit/risk profile, with no further details disclosed [113,143]. The data from the literature indicates promise in using SGN-2FF in cancer therapeutics however, the inhibitory potency requires further investigation [140]. 

### 6.4. De novo and Salvage Pathway Inhibition of Cellular Fucosylation 

New fucosylation inhibitors that act via the de novo and salvage pathways have been recently developed. The salvage pathway can be inhibited via two anomers of SGN-2FF (A2FF1P and B2FF1P). A2FF1P and B2FF1P enter the metabolic pathway at a later stage and have 4-7 times higher potency than SGN-2FF—believed to be due to better retainment inside the cell, and more efficient conversion of GDP-Fuc2F [140]. Pijnenborg et al. have also developed a class of fucose inhibitors that directly target *de novo* GDP-fucose biosynthesis. 90% of GDP-fucose is biosynthesized via *de novo* biosynthesis from GDP-mannose, thus direct inhibition could produce more potent inhibitors than the salvage pathway counterparts. Two new inhibitors, Fucotrim I (P-D-Rha6F2-1P) and Fucotrim II (P-D-Rha6F3-1P) are based on fluorinated mannose 1-phosphate derivatives, and have been shown to be more potent than SGN-2FF (which acts via the salvage pathway) [138]. The potency, specificty, and low toxicity of these inhibitors makes them exciting new candidates for cancer therapeutics.

## 7. Conclusions and Future Perspective

2020 saw the publication of several pivotal studies focussing on novel FUT8 discoveries. Key publications have ranged from the first in depth description of FUT8’s structural basis [43,44,144,145], identification of FUT8’s substrate specificity [146], its regulatory role amongst various cancers [27,82,147,148,149,150,151,152,153] and the inhibition of fucosylation [138,140]. The literature has vastly expanded upon our understanding and the particular importance of FUT8 in human disease. As FUT8 establishes itself as a critical component of cancer progression, the exciting potential to exploit FUT8 therapeutically will inevitably become an important focal point of the glyco-oncology field. Preclinical data of the FUT8 inhibitor SGN-2FF has demonstrated robust biological effects which have supported one clinical trial (NCT# 02952989). FUT8 is associated to some of the most aggressive and lethal cancers, supporting further the investigation into the effect of targeting FUT8 in combination with already approved antineoplastic drugs or immunotherapies to improve cancer therapy outcomes. The recent discoveries outlined here, have made considerable advancements in our understanding of FUT8’s fundamental biology. Using this information we can move forward, to realise the full potential of FUT8 as a critical factor in cancer diagnosis and therapeutics in the future.

## Figures and Tables

**Table 1:** Known fucosyltransferases in Humans. Adapted from [18,21] and created with GlycoGlyph [22].

Common Name(s)	Abbreviation	Subcellular Location [23]	Representative Major Products
H blood group α2fucosyltransferase	FUT1	Membrane	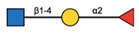
Secretor (Se) blood group α2fucosyltransferase	FUT2	Plasma membrane Cytosol	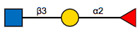
Fuc-TII α3/4fucosyltransferase Lewis blood group fucosyltransferase	FUT3	Intracellular membrane (different isoforms)	Sialyl-Lewis Structures 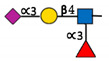 Lewis Structures 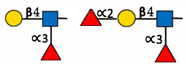
Fuc-TIV α3fucosyltransferase ELAM-1 ligand fucosyltransferase	FUT4	Vesicles	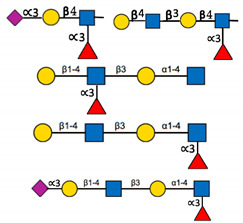
Fuc-TV α3fucosyltransferase	FUT5	Membrane	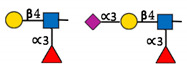
Fuc-VI α3fucosyltransferase	FUT6	Golgi apparatus	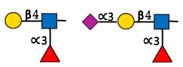
Fuc-VII α3fucosyltransferase	FUT7	Golgi apparatus	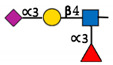
Fuc-VIII α3fucosyltransferase	FUT8	Golgi apparatus Cytosol	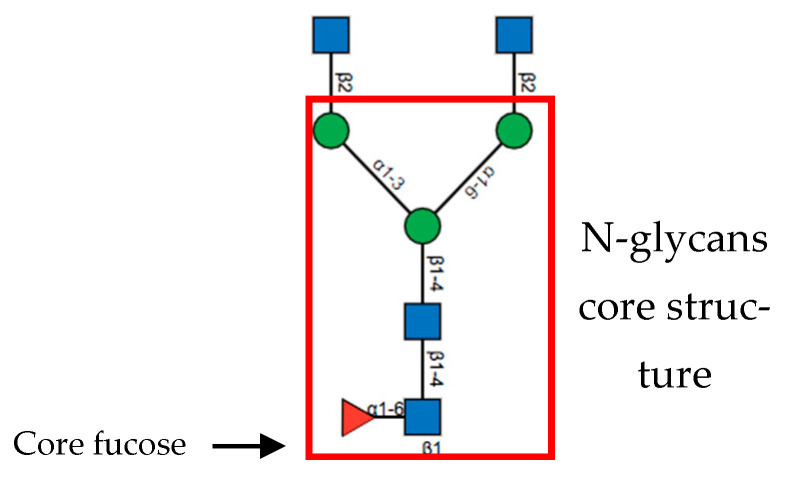
Fuc-TIX α3fucosyltransferase	FUT9	Nucleoplasm Endoplasmic reticulum Golgi apparatus	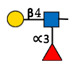
Fuc-TX α3fucosyltransferase	FUT10	Nucleoplasm Endoplasmic reticulum Golgi apparatus	Unknown
Fuc-TXI α3fucosyltransferase	FUT11	Nuclear membrane Golgi apparatus	Unknown
Protein O-fucosyltransferase 1	POFUT1/FUT12	Centrosome	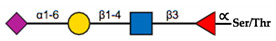
Protein O-fucosyltransferase 2	POFUT2/FUT13	Intracellular	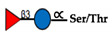

**Table 2:** FUT8 in cancer.

**Lung Cancer [20,28,49]**
FUT8 is upregulated in NSCLC and is associated with increased tumour metastasis, higher reoccurrence, and poorer survival. Core fucosylated E-cadherin hinders cell motility and reduction of fucosylation levels enhances lung cancer cell migration. Core fucosylation of E-cadherin regulates Src activation thereby inducing an EMT process—stimulating migration of lung cancer cells.
**Liver Cancer [50,52]**
Tumour tissue and highly metastatic HCC cells have high expression of FUT8. FUT8 is a direct transcriptional target of wild type p53 in HCC cells.
**Colorectal Cancer [24]**
FUT8 and E-cadherin levels are significantly increased in colorectal cancer.
**Ovarian Cancer [53]**
In epithelial ovarian cancer (EOC) alteration of core fucosylation is associated with cisplatin (cDDP) resistance. cDDP-uptake regulates cell survival, signal transduction, and cell apoptosis.
**Prostate Cancer [5,57]**
Increased core fucosylation in prostate cancer correlates to disease progression FUT8 is increased in high-grade and metastatic prostate cancers FUT8 overexpression may act as a driver in castrate-resistant phenotypes
**Breast Cancer [30]**
FUT8 is highly upregulated in aggressive breast carcinoma cells and plays a role in migratory and invasive capability and promotes distal lung metastasis
**Thyroid Cancer [56]**
FUT8 overexpression—specifically in cases with high biological aggressiveness. High FUT8 expression has significant association to tumour size, lymph node metastasis, advanced stage and presence of poorly differentiated lesions—indicating FUT8 expression is required for the progression of papillary carcinoma.
**Melanoma [55]**
FUT8 is important in melanoma invasion and represents a therapeutic target in melanoma metastasis. Silencing FUT8 reduces lung metastasis, and inhibits growth of pre-seeded metastases in the liver, brain and kidneys.
**Pancreatic Cancer [27]**
FUT8 protein expression is significantly elevated in carcinoma relative to normal pancreatic duct tissues. FUT8 is associated with lymph-node metastases and knockdown reduces cancer cell invasion.

## Data Availability

Data sharing not applicable.

## Abbreviations

ER	Endoplasmic reticulum
GALNT	N-acetylgalactosaminyltransferase
GalNAc	N-acetylgalactosamine
FUT8	α-(1,6)-fucosyltransferase
GLCNAc-TV or MGAT5	N-acetylglucosaminyltransferase
GDP-β-l-fucose	Guanosine diphosphate
GMD	GDP-mannose 4,6-dehydrastase
FX protein	GDP-keto-6-deoxymannose 3,5-epimerase, 4-reductase
NADPH	Nicotinamide adenine dinucleotide phosphate
AFP	α-fetoprotein
HCC	Hepatocellular carcinoma
LCA	Lens culinaris agglutinin
PCa	Prostate Cancer
PSA	Prostate Specific Antigen
BPH	Benign Prostate Hyperplasia
HPLC	High-performance liquid chromatography
AAL	*Aleuria aurantica* lectin
AGP	α1-acid glycoprotein
CAIE	Crossed affinoimmunoelectrophoresis
fAGP	α1,3fucosylated AGP
RPN1	Ribophorin I
FUTs	Fucosyltransferases
NSCLC	Nonsmall cell lung cancer
LEF-1	Lymphoid enhancer binding factor 1
CRC	Colorectal cancer
DFS	Disease free survival
EOC	Epithelial Ovarian Cancer
cDDP	Cisplatin
CTR1	Copper transporter 1
PrECs	Prostate epithelial cells
PrSCs	Prostate stromal cells
ADT	Androgen Deprivation Therapy
EV	Extracellular Vesicles
PDAC	Pancreatic ductal adenocarcinoma
ADCC	Antibody-dependent cellular cytotoxicity
PD-1	Programmed cell death
TGF-β	Transforming growth factor- β1
EGF	Epidermal growth factor
FcRs	Lymphocyte receptors
Fc	Constant region
IgG	Immunoglobulin G
CHO	Chinese hamster ovary
EMT	Epithelial-mesenchymal transition
E-box	Enhancer box
ZFNs	Zinc-finger nucleases
SGN-2FF	2-fluorofucose
FDA	US Food and Drug Administration
NHL	Non-Hodgkin’s lymphoma
CTCL	T-cell lymphoma
HTLV1	T-lymphotrophic virus 1
